# Special Issue “Research Progress of Bioimaging Materials”

**DOI:** 10.3390/ijms251910363

**Published:** 2024-09-26

**Authors:** Liangcan He

**Affiliations:** 1 Key Laboratory of Microsystems and Microstructures Manufacturing, School of Medicine and Health, Harbin Institute of Technology, Harbin 150001, China; liangcanhe@hit.edu.cn; 2 Zhengzhou Research Institute, Harbin Institute of Technology, Zhengzhou 450000, China

In the context of increasingly diverse diseases, early diagnosis and prevention, particularly in cancer control, have become more important than ever. Biomedical imaging technologies provide critical tools for early disease diagnosis, precise treatment, and biomedical research by enabling the real-time, non-invasive visualization of structural and functional changes within living organisms. This Special Edition aims to focus on the technological advancements of bioimaging materials in the fields of disease diagnosis and treatment, exploring their applications in unraveling the complex structures and dynamic processes of biological systems. These advancements are poised to facilitate drug development, personalized medicine, and the effective prevention of complex diseases, breaking through the current limitations of therapeutic approaches. Furthermore, this Special Issue also analyzes the challenges facing the research of bioimaging materials and provides insights into their future development directions.

Due to the complex structure of tissues, light is significantly scattered and absorbed by cells, blood vessels, and other tissue components during penetration, particularly in the short-wavelength range (such as visible light), leading to signal attenuation and blurring. Additionally, tissue autofluorescence generates high background noise, further reducing the signal-to-noise ratio. Deep tissue imaging also faces the challenges of phototoxicity and photobleaching, making long-term or high-intensity imaging more difficult. To overcome these challenges, there is a need for fluorescent probes with stronger penetration, higher signal-to-noise ratios, and minimal tissue damage. Deep red/near-infrared (NIR) fluorescent probes, which emit light in the deep red to NIR region, exhibit good tissue penetration and lower autofluorescence interference, making them particularly suitable for deep tissue imaging. They hold significant potential for biomedical applications such as tumor detection, vascular imaging, and organ observation. Eduardo et al. [1] explored the photophysical properties of COUPY dyes in the deep red and NIR regions by modifying the coumarin scaffold. They found that the introduction of strong electron-withdrawing groups and the julolidine ring structure significantly improved the dyes’ photostability, imaging performance, and enhanced mitochondrial specificity, offering new insights for designing effective bioimaging probes. Hyeok Lee et al. [2] developed a novel NIR fluorescent probe, NIR-HCy-NO_2_, which incorporates a nitro group into the hemicyanine scaffold, enabling the efficient detection of nitroreductase (NTR) activity. This probe exhibited low background noise and high fluorescence enhancement and showed significant potential for in vivo cell imaging, particularly in interactions with Type I mitochondrial NTR.

The cell membrane plays a crucial role in communication between cells and their external environment or neighboring cells, and changes in the membrane can affect cellular properties. However, tracking dynamic changes in the membranes of living cells remains challenging, especially during long-term studies under detachment conditions in tissue regeneration and cancer metastasis. Rzewnicka et al. [3] introduced a new dithienothiophene S,S-dioxide (DTTDO) derivative that effectively stains live cell membranes, retaining its staining capability even under detachment conditions. This dye, with its excellent optical properties and bioactivity, is suitable for the long-term observation of membrane dynamics, such as during cell migration, division, and fusion, with reduced background signal, making it valuable for studies of tissue regeneration and cancer metastasis.

Microorganisms have important applications in bioimaging due to their unique metabolic activities and genetic engineering modifications, allowing them to express fluorescent proteins or other visualizable molecules. These modified microorganisms can be used for in vivo imaging to track microbial distribution and behavior within organisms or to detect specific pathological processes or biological signals. Leveraging the ability of bacteria to target specific tissues or microenvironments, particularly in tumors and hypoxic regions, bacterial imaging materials can be used for the early diagnosis and tracking of diseases. Lim et al. [4] constructed a recombinant plasmid, pRFN4-Pp luxLP-N-lumP, by introducing the N-lumP gene from marine bioluminescent bacteria and the lux promoter into Escherichia coli, significantly enhancing the fluorescence intensity, achieving a fluorescence strength 500 times that of regular E. coli. This fluorescent bacterial system, utilizing riboflavin and lux genes, offers a new tool for high-sensitivity biosensor applications. Moreover, bacteria can be designed to synthesize optical nanomaterials, providing a multifunctional imaging platform. This expands the possibilities of in vivo imaging technologies and promotes the integration of diagnostics and therapeutics.

Inorganic fluorescent probes, such as magnetic nanoparticles, upconversion nanoparticles (UCNPs), and rare-earth-doped materials, are gaining attention in high-resolution imaging due to their unique optical properties. Rare-earth-doped nanomaterials, with their distinctive optical characteristics and good biocompatibility, significantly improve imaging sensitivity and signal intensity, making them especially suitable for deep tissue imaging. Xiao et al. [5] incorporated Ce^3+^ ions into Cs_3_Bi_2_Cl_9_ lattices using an improved antisolvent method, significantly increasing the photoluminescence quantum yield of bismuth-based perovskite quantum dots to 22.12%. These materials also exhibited excellent water solubility, biocompatibility, and enhanced upconversion fluorescence, offering a new strategy for applying perovskites in bioimaging. Furthermore, rare-earth-doped materials possess multimodal imaging capabilities, combining fluorescence, magnetic resonance, and other imaging techniques to provide more accurate diagnostics. These materials also have the potential for theranostics, paving the way for early disease diagnosis and personalized treatment.

Theranostic materials enable the early detection of diseases and the immediate initiation of treatment. At the early stages of a disease, probes or drug carriers can detect abnormalities through imaging functions while simultaneously releasing therapeutic drugs to intervene, preventing further disease progression. This is especially effective in treating major diseases such as cancer and cardiovascular conditions. Dong et al. [6] used cobalt-doped carbon dots (CoNCDs) to generate reactive oxygen species (ROS) that modulate the tumor microenvironment and combined them with a cGAS-STING agonist to successfully remodel the tumor microenvironment, synergistically enhancing antitumor efficacy, offering a promising approach for combined antitumor therapy. Li et al. [7] developed a 4′-substituted-2,2′:6′,2′-terpyridine complex containing oxygen substituents, showing superior antitumor activity compared to cisplatin, with its anticancer effects closely related to its enhanced DNA-binding ability, providing a theoretical basis for designing novel antitumor terpyridine metal complexes. Dai et al. [8] designed PSMA antibody-modified sonosensitive nanoparticles (PFP@IR780@PTX@liposome NPs), which, upon low-intensity focused ultrasound (LIFU) induction, undergo a liquid–gas phase transition, specifically accumulating in prostate tumors, thus facilitating visualization and enhanced chemotherapy. This nanoplatform controlled the release of docetaxel and monitored therapy via ultrasound and photoacoustic imaging, offering a novel approach to imaging and therapy in the field of bioimaging materials.

siRNA gene therapy has the potential for long-term treatment by regulating gene expression to treat cancers, cardiovascular diseases, and degenerative disorders. However, due to siRNA’s susceptibility to degradation by nucleases, appropriate carriers are required to enhance its stability and efficacy. Non-viral carriers, favored for their low immunogenicity and high safety, have become the mainstream choice. Tong et al. [9] reviewed common non-viral carriers in recent years, their advantages and disadvantages, and the latest applications. Bioimaging materials, as siRNA carriers, not only allow the real-time monitoring of delivery and distribution but also enhance the precision and efficacy of gene therapy by visualizing the targeting process, reducing side effects and improving safety. He et al. [10] first synthesized hetero-assembled gold–silver nanoclusters (Au-Ag NCs), demonstrating their potential as high-efficiency carriers. This porous structure exhibited excellent drug release performance in simulated tumor environments and could be controlled in size and morphology through ultrasound, making it a smart carrier for drug and siRNA delivery.

Biocompatibility is also a critical prerequisite for the application of bioimaging materials. Prolonged accumulation of materials in the body may lead to adverse reactions, necessitating the design of degradable or excretable bioimaging materials to minimize immune responses and toxicity. Górecka et al. [11] explored the long-term in vitro evaluation of polycaprolactone (PCL)-based composites, observing changes in properties such as water contact angle, stiffness, and radiopacity during a 24-week degradation. Their research aids in screening composites suitable for FM manufacturing, providing important guidance for their application in bioimaging.

The design of bioimaging materials must balance good biocompatibility with high signal intensity to ensure the precise detection of target molecules or cells within living organisms. The functionalization of these materials is equally important, allowing for specific targeted imaging through surface modification with targeting molecules. One of the main challenges in current bioimaging material research is how to maintain high sensitivity while minimizing toxicity. Additionally, high production costs and complex synthesis processes limit their widespread use. Future research will focus on the development of smart materials, integration of controlled-release systems, and optimization of multimodal imaging technologies. By promoting interdisciplinary integration across material science, biology, medicine, and computational science, it is hoped that more efficient and accurate bioimaging materials can be created, enabling early disease detection, precise treatment, and treatment monitoring, ultimately improving therapeutic outcomes while reducing side effects.

## Data Availability

Not applicable.
